# Supplementary material for: FRMD7 Gene Alterations in a Pakistani Family Associated with Congenital Idiopathic Nystagmus
Source: Genes (Basel). 2023 Jan 29;14(2):346. doi: 10.3390/genes14020346 (PMC9957179; doi:10.3390/genes14020346)
Supplement: Supplementary file 1 [file genes-14-00346-s001.zip › Supplementary Table S1.docx]

**Supplementary Table S1** Primer sequences used for Co-segregation analysis of family PKNYS07

| **Primer** | **Sequence** | **GC content** | **Amplicon size** | **Melting Temperature (T_m_)** |
| --- | --- | --- | --- | --- |
| FRMD7_c.443T>A_F | ATCTCAGCGTTTCATGGAGC | 45% | 237 bp | 57°C |
| FRMD7_c.443T>A_R | TGCAGCAGAACTTGGAGACT | 45% | 237 bp | 57°C |
