# Supplementary material for: FRMD7 Gene Alterations in a Pakistani Family Associated with Congenital Idiopathic Nystagmus
Source: Genes (Basel). 2023 Jan 29;14(2):346. doi: 10.3390/genes14020346 (PMC9957179; doi:10.3390/genes14020346)
Supplement: Supplementary file 1 [file genes-14-00346-s001.zip › Supplementary Table S2.docx]

**Supplementary Table S2** Loci associated with nystagmus phenotype

| **Locus** | **OMIM ***  **ID** | **MOI**** | **Gene**  **Detected** | **Reference** |
| --- | --- | --- | --- | --- |
| NYS 1 (Xq26.2) | 310700 | X - Linked | Yes (*FRMD7*) | [[19](#_ENREF_19)] |
| NYS 2 (6p12) | 164100 | Autosomal Dominant | No | [[42](#_ENREF_42)] |
| NYS 3 (7p11.2) | 608345 | Autosomal Dominant | No | [[3](#_ENREF_3)] |
| NYS 4 (13q31-33) | 193003 | Autosomal Dominant | No | [[43](#_ENREF_43)] |
| NYS 5 (Xp11.4) | 300589 | X - Linked | No | [[7](#_ENREF_7)] |
| NYS 6 (Xp22.2) | 300814 | X - Linked | Yes (*GPR143*) | [[9](#_ENREF_9)] |
| NYS 7 ([1q31.3-q32.1](https://www.omim.org/geneMap/1/1413?start=-3&limit=10&highlight=1413)) | 614826 | Autosomal Dominant | No | [[44](#_ENREF_44)] |
