# Supplementary material for: FRMD7 Gene Alterations in a Pakistani Family Associated with Congenital Idiopathic Nystagmus
Source: Genes (Basel). 2023 Jan 29;14(2):346. doi: 10.3390/genes14020346 (PMC9957179; doi:10.3390/genes14020346)
Supplement: Supplementary file 1 [file genes-14-00346-s001.zip › Supplementary Table S3.docx]

**Supplementary Table S3.** List of currently reported mutations in the *FRMD7* gene [Source: HGMD^®^ professional]

| **Mutation type** | **Codon Change** | **Amino acid change** | **Nucleotide** | **Protein** | **Phenotype** | **Reference** |
| --- | --- | --- | --- | --- | --- | --- |
| Missense/  Nonsense | ATG-GTG | Met1Val | c.1A> G | p.M1V | IN | [[45](#_ENREF_45)] |
|  | TTT-TCT | Phe16Ser | c.47T>C | p.F16S | NYS1 | [[46](#_ENREF_46)] |
|  | CAA-TAA | Gln20Term | c.58C>T | p.Q20* | NYS1 | [[12](#_ENREF_12),[47](#_ENREF_47)] |
|  | GGG-AGG | Gly24Arg | c.70G>A | p.G24R | NYS 1 | [[19](#_ENREF_19),[48](#_ENREF_48)] |
|  | GGG-GAG | Gly24Glu | c.71G>A | p.G24E | NYS1 | [[19](#_ENREF_19),[31](#_ENREF_31)] |
|  | GGG-TGG | Gly24Trp | c.70G>T | p.G24W | IN | [[49](#_ENREF_49)] |
|  | CGA-TGA | Gly52Term | c.154G>T | p.G52* | IN | [[29](#_ENREF_29)] |
|  | CTA-CGA | Leu57Arg | c.170 T>G | p.L57R | NYS1 | [[28](#_ENREF_28)] |
|  | GAC-TAC | Asp85Tyr | c.253G>T | p.D85Y | NYS1 | [[28](#_ENREF_28)] |
|  | TCA-CCA | Ser123Pro | c.367 T>C | p.S123P | NYS1 | [[28](#_ENREF_28)] |
|  | CTG-CGG | Leu142Arg | c.425T>G | p.L142R | NYS1 | [[19](#_ENREF_19)] |
|  | CGG-TGG | Arg146Trp | c.436C>T | p.R146W | NYS1 | [[50](#_ENREF_50)] |
|  | ATA-AAA | Ile158Asn | c.473T>A | p.I158N | NYS | [[6](#_ENREF_6)] |
|  | ATG-GTG | Met186Val | c.556A>G | p.M186V | NYS | [[51](#_ENREF_51)] |
|  | CAT-CCT | His192Pro | c.575A>C | p.H192P | IN | [[52](#_ENREF_52)] |
|  | GCC-ACC | Ala194Thr | c.580G>A | p.A194T | IN | [[32](#_ENREF_32)] |
|  | GAC-TAC | Asp196Tyr | c.586G>T | p.D196Y | NYS1 | [[28](#_ENREF_28)] |
|  | CAG-TAG | Gln201Term | c.601C>T | p.Q201* | NYS1 | [[19](#_ENREF_19),[47](#_ENREF_47)] |
|  | ATA-AAA | Ile202Asn | c.605T>A | p.I202N | NYS | [[6](#_ENREF_6)] |
|  | GTC-ATC | Val206Ile | c.616G>A | p.V206I | NYS1 | [[28](#_ENREF_28)] |
|  | CAC-CGC | His208Arg | c.623A>G | p.H208R | IN | [[53](#_ENREF_53)] |
|  | GGA-GCA | Gly210Arg | c.628G>C | p.G210R | NYS1 | [[28](#_ENREF_28)] |
|  | CTG-CCG | Leu212Pro | c.635T>C | p.L212P | XCIN | [[54](#_ENREF_54)] |
|  | AAG-AAT | Lys219Asn | c.657G>T | p.K219N | NYS1 | [[46](#_ENREF_46)] |
|  | AAT-GAT | Asn221Asp | c.661A>G | p.N221D | NYS1 | [[19](#_ENREF_19)] |
|  | TGG-CGG | Trp225Arg | c.673T>C | p.W225R | IN | [[29](#_ENREF_29)] |
|  | TGG-GGG | Trp225Gly | c.673T>G | p.W225G | NYS1 | [[12](#_ENREF_12)] |
|  | GCT-ACT | Ala226Thr | c.676G>A | p.A226T | NYS1 | [[19](#_ENREF_19)] |
|  | CGC-TGC | Arg229Cys | c.685C>T | p.R229C | NYS1 | [[31](#_ENREF_31),[50](#_ENREF_50)] |
|  | CGC-GGC | Arg229Gly | c.685C>G | p.R229G | NYS | [[27](#_ENREF_27)] |
|  | CGA-CAA | Arg299His | c.686G>A | p.R229H | NYS1 | [[28](#_ENREF_28)] |
|  | TTG-GTG | Leu231Val | c.691T>G | p.L231V | NYS 1 | [[19](#_ENREF_19)] |
|  | ATC-ACC | Ile240Thr | c.719T>C | p.I240T | XCIN, | [[55](#_ENREF_55)] |
|  | AAGAGG | Lys241Arg | c.722A>G | p.K241R | IN | [[52](#_ENREF_52)] |
|  | ATG-ACG | Met258Thr | c.773 T>C | p.M258T | NYS1 | [[28](#_ENREF_28)] |
|  | AGCAGA | Ser260Arg | c.780C>A | p.S260R | XCIN | [[56](#_ENREF_56)] |
|  | CGA-CAA | Arg261Gln | c.782G>A | p.R261Q | IN | [[49](#_ENREF_49)] |
|  | CGA-GGA | Arg261Gly | c.781C>G | p.R261G | NYS1 | [[50](#_ENREF_50),[57](#_ENREF_57)] |
|  | CGA-TGA | Arg261Term | c.781C>T | p.R261* | NYS1 | [[28](#_ENREF_28)] |
|  | GCT-CCT | Ala266Pro | c.796G>C | p.A266P | NYS1 | [[19](#_ENREF_19),[29](#_ENREF_29)] |
|  | TTC-TTA | Phe267Leu | c.801C>A | p.F267L | IN | [[48](#_ENREF_48)] |
|  | TGG-TGT | Trp268Cys | c.804G>T | p.W268C | NYS1 | [[46](#_ENREF_46)] |
|  | GGA-GGC | Lys269Gln | c.805 A > C | p.K269Q | NYS | [[58](#_ENREF_58)] |
|  | TGT-TTT | Cys271Phe | c.812G>T | p.C271F | NYS1 | [[33](#_ENREF_33),[49](#_ENREF_49)] |
|  | TGT-AGT | Cys271Ser | c.811T>A | p.C271S | NYS | [[59](#_ENREF_59)] |
|  | TGT-TAT | Cys271Tyr | c.812G>A | p.C271Y | NYS1 | [[19](#_ENREF_19),[29](#_ENREF_29),[31](#_ENREF_31)] |
|  | CAT-CGT | His275Arg | c.824A>G | p.H275R | IN | [[29](#_ENREF_29)] |
|  | CAT-CCT | His275Pro | c.824A>C | p.H275P | NYS1 | [[12](#_ENREF_12)] |
|  | GAG-GAC | Glu238Asp | c.849G>C | p.E283D | NYS1 | [[28](#_ENREF_28)] |
|  | CTC-CCC | Leu292Pro | c.875T>C | p.L292P | IN | [[48](#_ENREF_48)] |
|  | GGT-CGT | Gly296Arg | c.886G>C | p.G296R | NYS1 | [[48](#_ENREF_48),[50](#_ENREF_50)] |
|  | GGC-TGC | Gly296Cys | c.886G>T | p.G296C | IN | [[60](#_ENREF_60)] |
|  | GGA-CGA | Gly296Arg | c.887G>C | p.G296R | NYS1 | [[28](#_ENREF_28)] |
|  | GGC-GAC | Gly296Asp | c.887G>A | p.G296D | NYS1 | [[28](#_ENREF_28)] |
|  | GGC-GTC | Gly296Val | c.887G>T | p.G296V | NSY1 | [[28](#_ENREF_28)] |
|  | TAT-TGT | Tyr301Cys | c.902A>G | p.Y301C | NYS1 | [[19](#_ENREF_19)] |
|  | CGA-TGA | Arg304Term | c.910C>T | p.R304* | IN | [[47-49](#_ENREF_47)] |
|  | CAA-CGA | Gln306Arg | c.917A>G | p.Q306R | NYS | [[61](#_ENREF_61)] |
|  | CAA-AAA | Gln208Lys | c.922C>A | p.Q308K | NYS1 | [[28](#_ENREF_28)] |
|  | AGA-GGA | Arg325Gly | c.973A>G | p.R325G | IN | [[32](#_ENREF_32)] |
|  | CGG-CAG | Arg325Lys | c.974G>A | p.R325K | NYS1 | [[28](#_ENREF_28)] |
|  | TAC-TGC | Tyr328Cys | c.983A>G | p.Y328C | IN | [[29](#_ENREF_29)] |
|  | CCA-CAA | Pro329Gln | c.986C>A | p.P329Q | NYS | [[57](#_ENREF_57)] |
|  | CGA-TGA | Arg335Term | c.1003C>T | p.R335* | NYS1 | [[19](#_ENREF_19),[29](#_ENREF_29),[62](#_ENREF_62)] |
|  | TCA-TTA | Ser340Leu | c.1019C>T | p.S340L | NYS1 | [[19](#_ENREF_19),[31](#_ENREF_31)] |
|  | TAT-TAG | Tyr358Term | c.1074T>G | p.Y358* | NYS1 | [[28](#_ENREF_28)] |
|  | CAA-TAA | Gln364Term | c.1090C>T | p.Q364* | IN | [[63](#_ENREF_63)] |
|  | CAG-TAG | Gln487Term | c.1459C>T | p.Q487* | XCIN | [[56](#_ENREF_56)] |
|  | TGG-TGA | Trp508Tern | c.1523G>A | p.W508* | NYS1 | [[28](#_ENREF_28)] |
|  | TGG-TGA | Trp508Term | c.1524G>A | p.W508* | NYS1 | [[46](#_ENREF_46)] |
| Splicing |  |  | c.57+1G>A |  | NYS | [[6](#_ENREF_6)] |
|  |  |  | c.57+5G>A |  | NYS | [[12](#_ENREF_12)] |
|  |  |  | c.57+2 T>C |  | NYS1 | [[28](#_ENREF_28)] |
|  |  |  | c.58-3 T>A |  | NYS1 | [[28](#_ENREF_28)] |
|  |  |  | c.58-1G>A |  | NYS | [[47](#_ENREF_47),[59](#_ENREF_59)] |
|  |  |  | c.162+5G>A |  | NYS1 | [[19](#_ENREF_19),[47](#_ENREF_47)] |
|  |  |  | c.162+6T>C |  | IN | [[52](#_ENREF_52)] |
|  |  |  | c.163-1G>T |  | NYS | [[64](#_ENREF_64)] |
|  |  |  | c.205+2T>G |  | NYS1 | [[19](#_ENREF_19),[29](#_ENREF_29),[47](#_ENREF_47)] |
|  |  |  | c.206-2 T>G |  | NYS1 | [[28](#_ENREF_28)] |
|  |  |  | c.206-5T>A |  | IN | [[29](#_ENREF_29)] |
|  |  |  | c.252G>A |  | NYS1 | [[19](#_ENREF_19),[47](#_ENREF_47)] |
|  |  |  | c.284+1G>A |  | NYS1 | [[19](#_ENREF_19),[47](#_ENREF_47)] |
|  |  |  | c.285-118C>T |  | IN | [[29](#_ENREF_29)] |
|  |  |  | c.497+5G>A |  | IN | [[48](#_ENREF_48)] |
|  |  |  | c.498-2A>G |  | IN | [[12](#_ENREF_12)] |
|  |  |  | c.498-2A>T |  | NYS1 | [[28](#_ENREF_28)] |
|  |  |  | c.645+1delinsAT |  | NYS1 | [[28](#_ENREF_28)] |
|  |  |  | c.645+1G>C |  | IN | [[19](#_ENREF_19)] |
|  |  |  | c.974+2 T>C |  | NYS1 | [[28](#_ENREF_28)] |
|  |  |  | c.1050+1G>C |  | IN | [[19](#_ENREF_19),[47](#_ENREF_47)] |
|  |  |  | c.1050+5G>A |  | IN | [[47](#_ENREF_47),[65](#_ENREF_65)] |
| Small  Deletions |  |  | c.41_43delAGA | p.K14del | NYS1 | [[19](#_ENREF_19)] |
|  |  |  | c.660delC | p.Asn221Ilefs*11 | IN | [[29](#_ENREF_29),[48](#_ENREF_48)] |
|  |  |  | c.694_695delAG | p.S232FfsX233 | IN | [[49](#_ENREF_49)] |
|  |  |  | c.823-829delACCCTAC | p.Thr275fs | IN | [[66](#_ENREF_66)] |
|  |  |  | c.887delG | p.G296VfsX318 | NYS1 | [[19](#_ENREF_19)] |
|  |  |  | c.980_983delATTA | p.H327PfsX353 | NYS | [[57](#_ENREF_57)] |
|  |  |  | c.1241delT | p.F414Sfs*30 | NYS1 | [[28](#_ENREF_28)] |
|  |  |  | c.1262delC | p.P421LfsX443 | NYS1 | [[19](#_ENREF_19)] |
|  |  |  | c.1275_1276delTG | p.E426AfsX429 | NYS1 | [[33](#_ENREF_33)] |
|  |  |  | c.1492delT | p.F495fs24X | NYS1 | [[46](#_ENREF_46)] |
|  |  |  | c.1489-1492delTTTT | p.F497fs26X | NYS1 | [[67](#_ENREF_67)] |
|  |  |  | c.1645delG | p.V549YfsX554 | XCIN | [[56](#_ENREF_56)] |
|  |  |  | c.1860_1861 delAG | p.D621Pfs*27 | NYS1 | [[28](#_ENREF_28)] |
|  |  |  | c.1918delA | p.S640fs | NYS1 | [[28](#_ENREF_28)] |
|  |  |  | c.2036delT | p.Leu679Argfs*8 | IN | [[48](#_ENREF_48)] |
| Gross  Deletions |  |  | 1.29 Mb incl. entire gene + 6 others |  | IN | [[48](#_ENREF_48)] |
|  |  |  | 1.29 mb incl. entire gene + IGSF1 + 5 others |  | NYS | [[68](#_ENREF_68)] |
|  |  |  | incl ex. 2-4 |  | NYS1 | [[69](#_ENREF_69)] |
|  |  |  | Exon1 |  | NSY1 | [[28](#_ENREF_28)] |
| Insertions |  |  | c.478_479insT | p.F161LfsX172 | NYS1 | [[19](#_ENREF_19)] |
|  |  |  | c.880dupA | p.S294KfsX302 | NYS1 | [[13](#_ENREF_13)] |
|  |  |  | c.998dupA | p.His333Glnfs*2 | IN | [[32](#_ENREF_32)] |
|  |  |  | c.1419_1422dup | p.Tyr475fs | IN | [[70](#_ENREF_70)] |
|  |  |  | c.1442_1443 insAT | p.P482Ffs*43 | NSYS1 | [[28](#_ENREF_28)] |
|  |  |  | c.1493insA | p.Y498X | NYS | [[6](#_ENREF_6)] |

IN= Idiopathic Nystagmus; NYS= Nystagmus, XCIN= X linked congenital idiopathic Nystagmus
